# “Unifying the Profession” in the Interest of the Great Medical Journal Trust—Proselyting the Pathies

**Published:** 1907-03

**Authors:** 


### EDITORIAL DEPARTMENT.

### "UNIFYING THE PROFESSION" IN THE INTEREST OF THE GREAT MEDICAL JOURNAL TRUST —PROSELYTING THE PATHIES.

It will be remembered that he who holds the destiny of the American medical profession in his hands (or thinks he does, or would if he could)—one Simmons—of The Octopus, is an apostate homeopath. He having abjured the faith of Hahnemanism, wants all others to do so—to keep him in countenance, as it were, like the fox who got his tail cut off, you know. To this end he has conceived the idea of a campaign and has launched a propaganda—a crusade of proselytism by paid emissaries — for that purpose, really, but ostensibly to "unify the profession," by coaxing or driving into the fold of the State and, *ergo,* of the National organization all of the physicians of America who, for reasons of their own, have held back, so far, and do not want to join. Pressure is to be applied to get them into the bull ring. Whether they want to or not; they have just got to, or be boycotted.

The States are swarming with these emissaries—paid by the Octopus. There are five of them now in Texas, and five in Missouri, so we are informed by one of them who blew into Austin recently and addressed the Travis County Medical Society, or, rather, nine members (of fifty-four) who went to hear him. It came out during his remarks that in addition to rounding up and checking off all the regulars eligible to membership but who are not of the elect, the orders are to round up the homeopaths and other "paths" as well, and get them to "jine in the good work" of unifying the profession to the end that McCormack's work may be perfected, and all power placed in the hands of the Octopus. When that glorious day shall come; when everything called "doctor" shall pay tribute to the Octopus and pour shekels into the coffer of the great *J. A. M. A.,* where, oh, where will be the independent medical press—the "individually-owned" and "conducted-for-profit" medical journal? Like Bozarris who, "in his guarded tent was dreaming of the hour when Greece with knee in suppliance bent should tremble at his power," the autocrat of the Octopus is doubtless dreaming of the hour when the *New York Medical Record,* the *New York Medical Journal,* the *Medical*

*Times,* the *Charlotte, N. C.,* the *New England Medical Journal,* the *Southern Practitioner,* the *Medical Sentinel,* the *Pacific,* and the little "Red Back" and all the others shall turn up their toes to the daisies. If he can get the homeos to do as he did, —go back on their raisin', and all "jine in" to swell the chorus of Glory to the Highest; to the great Octopus, it will be a happy day for the Dictator, and a *cold* day for certain pugnacious Independents who have made it *hot* for him.

And the funny part of it is that county societies are falling in, or down—before this ukase; but the homeos, I learn, are laughing in their sleeves and saying "I smell a mice." The Dictator of this councillor district is right in for it. He says, "We are working under a new dispensation, doctor"—(to me and in the county society meeting). "Yes," I said, "it may be a new dispensation, but you can't 'dispense' with our Principles of Medical Ethics which makes them quacks, and not in 'honorable standing,' nor can you 'dispense' with the resolution adopted by the Texas State Medical Society at Dallas in 1896 declaring that 'this Association will expel from membership any one who consults with a homeopath." By the bye, Jenkins of Brown county, in arguing against the one board bill—a consolidating of regulars, homeos, *osteos,* and physiomeds, and eclectics, in the House last week, read this resolution, and denounced the combine in the language of William Lloyd Garrison, who said in the U. S. Senate that slavery was a "league with death and a covenant with hell." He made some of our fellows feel like three 10-cent pieces, i. e., 30 cents. McGregor also read an editorial ridiculing the osteopaths, in the State Association journal of November, 1905, reproduced herewith, which sounded funny now that the Association has embraced them. Fancy an osteopath being licensed by "regulars" to treat infectious diseases by massage alone.

Now, county medical associations countenance and welcome emissaries from the Boss in control of the Octopus, and aid in the laudable cause of proselyting and bringing into the bosom of regular medicine these lost sheep. Oh, Consistency!

To my mind it is very clear that the purpose is to "unify the profession"—to mix fish, flesh, fowl and red herring into one heterogeneous mass, or mess, for the support of the Octopus and its feeders, the State Association journals; a medical journal trust, to crush out independent action.

What will the editors of the 200 independent, personally-owned, run-for-profit journals do about it? They meet in June. We will see. Be ye *men,* and submit to the Boss tyranny that would crush you out?

